# NSs Protein of Sandfly Fever Sicilian Phlebovirus Counteracts Interferon (IFN) Induction by Masking the DNA-Binding Domain of IFN Regulatory Factor 3

**DOI:** 10.1128/JVI.01202-18

**Published:** 2018-11-12

**Authors:** Jennifer Deborah Wuerth, Matthias Habjan, Julia Wulle, Giulio Superti-Furga, Andreas Pichlmair, Friedemann Weber

**Affiliations:** a Institute for Virology, Philipps-University Marburg, Marburg, Germany; b Institute for Virology, FB10-Veterinary Medicine, Justus-Liebig University, Giessen, Germany; c Innate Immunity Laboratory, Max Planck Institute of Biochemistry, Munich, Germany; d CeMM Research Center for Molecular Medicine, Austrian Academy of Sciences, Vienna, Austria; e Center for Physiology and Pharmacology, Medical University of Vienna, Vienna, Austria; f Institute of Virology, School of Medicine, Technical University of Munich, Munich, Germany; g Munich Partner Site, German Center for Infection Research (DZIF), Munich, Germany; Icahn School of Medicine at Mount Sinai

**Keywords:** DNA-binding domain, IRF3, NSs, sandfly fever Sicilian virus, interferon beta promoter, interferon induction

## Abstract

Sandfly fever Sicilian virus (SFSV) is one of the most widespread and frequently identified members of the genus *Phlebovirus* (order *Bunyavirales*, family *Phenuiviridae*) infecting humans. Being transmitted by *Phlebotomus* sandflies, SFSV causes a self-limiting, acute, often incapacitating febrile disease (“sandfly fever,” “Pappataci fever,” or “dog disease”) that has been known since at least the beginning of the 20th century. We show that, similarly to other pathogenic phleboviruses, SFSV suppresses the induction of the antiviral type I interferon (IFN) system in an NSs-dependent manner. SFSV NSs interfered with the TBK1-interferon regulatory factor 3 (IRF3) branch of the RIG-I signaling pathway but not with NF-κB activation. Consistently, we identified IRF3 as a host interactor of SFSV NSs. In contrast to IRF3, neither the IFN master regulator IRF7 nor any of the related transcription factors IRF2, IRF5, and IRF9 were bound by SFSV NSs. In spite of this specificity for IRF3, NSs did not inhibit its phosphorylation, dimerization, or nuclear accumulation, and the interaction was independent of the IRF3 activation or multimerization state. In further studies, we identified the DNA-binding domain of IRF3 (amino acids 1 to 113) as sufficient for NSs binding and found that SFSV NSs prevented the association of activated IRF3 with the IFN-β promoter. Thus, unlike highly virulent phleboviruses, which either destroy antiviral host factors or sequester whole signaling chains into inactive aggregates, SFSV modulates type I IFN induction by directly masking the DNA-binding domain of IRF3.

**IMPORTANCE** Phleboviruses are receiving increased attention due to the constant discovery of new species and the ongoing spread of long-known members of the genus. Outbreaks of sandfly fever were reported in the 19th century, during World War I, and during World War II. Currently, SFSV is recognized as one of the most widespread phleboviruses, exhibiting high seroprevalence rates in humans and domestic animals and causing a self-limiting but incapacitating disease predominantly in immunologically naive troops and travelers. We show how the nonstructural NSs protein of SFSV counteracts the upregulation of the antiviral interferon (IFN) system. SFSV NSs specifically inhibits promoter binding by IFN transcription factor 3 (IRF3), a molecular strategy which is unique among phleboviruses and, to our knowledge, among human pathogenic RNA viruses in general. This IRF3-specific and stoichiometric mechanism, greatly distinct from the ones exhibited by the highly virulent phleboviruses, correlates with the intermediate level of pathogenicity of SFSV.

## Abstract (precis)

Phleboviruses are receiving increased attention due to the constant discovery of new species and the ongoing spread of long-known members of the genus. Outbreaks of sandfly fever were reported in the 19th century, during World War I, and during World War II. Currently, SFSV is recognized as one of the most widespread phleboviruses, exhibiting high seroprevalence rates in humans and domestic animals and causing a self-limiting but incapacitating disease predominantly in immunologically naive troops and travelers. We show how the nonstructural NSs protein of SFSV counteracts the upregulation of the antiviral interferon (IFN) system. SFSV NSs specifically inhibits promoter binding by IFN transcription factor 3 (IRF3), a molecular strategy which is unique among phleboviruses and, to our knowledge, among human pathogenic RNA viruses in general. This IRF3-specific and stoichiometric mechanism, greatly distinct from the ones exhibited by the highly virulent phleboviruses, correlates with the intermediate level of pathogenicity of SFSV.

## INTRODUCTION

Members of the genus phlebovirus (order Bunyavirales, family *Phenuiviridae*) are present worldwide and gain increasing attention as vector-borne agents of disease (1). In addition to prominent, recently emerged phleboviruses such as severe fever with thrombocytopenia syndrome virus (SFTSV) in Asia and Heartland virus (HRTV) in North America (2), there are long-known members, such as Rift Valley fever virus (RVFV), Punta Toro virus (PTV), Toscana virus (TOSV), and sandfly fever Sicilian virus (SFSV), that are often reemerging or spreading into new geographical areas (3). In addition to these highly virulent (SFTSV, HRTV, and RVFV) and intermediately virulent (TOSV and SFSV) human pathogens, rapid progress in high-throughput sequencing enabled the identification of novel phleboviruses for which the disease potential is either recognized (e.g., sandfly fever Turkey virus [4] and Adria virus [5]) or not yet clarified (e.g., Massilia virus [6], Aguacate virus [7], and Dashli virus [8]).

Infection by SFSV and related sandfly fever viruses, all transmitted by phlebotomine sandflies, typically presents as an acute febrile disease with abrupt onset, often developing into incapacitating myalgia, headaches, malaise, leukocytopenia, or ocular or gastrointestinal symptoms (9, 10). An outbreak of this so-called “sandfly fever,” “Pappataci fever,” or “dog disease” during the Sicilian campaign of World War II in 1943 enabled Albert Sabin to isolate SFSV from infected soldiers (11). SFSV later proved to be one of the most widespread phleboviruses; it is present across the entire Mediterranean basin, in Portugal, in the Middle East inclusive of the Arabian peninsula, in Sudan, in Ethiopia, and in Somalia and in locations as distant as India and Bangladesh (12–18). In regions of endemicity, seroprevalence can reach levels of up to 50% in humans and close to 80% in dogs and other domestic animals, including cattle (12, 14, 19, 20). Hence, sandfly fever viruses are recognized as a significant public health threat, predominantly for immunologically naive groups such as soldiers or travelers (21–24). Nonetheless, little is known about the molecular interplay of SFSV and SFSV-like viruses with the host organism.

Like all phleboviruses, SFSV contains a tripartite single-stranded RNA genome (1, 3). While the large (L) genome segment and the medium (M) genome segment encode the viral polymerase (Pol) L and the glycoproteins, respectively, in a negative orientation, the small (S) segment codes for the nucleocapsid protein N and the nonstructural protein NSs in an ambisense manner. The genomic RNA segments are packaged into ribonucleoproteins (RNPs) by the nucleocapsid N protein and the L polymerase and are transcribed and replicated in the cytoplasm (25).

Due to complementarity of the 5ʹ and 3ʹ termini, the three RNP-packaged genome segments have the capacity to anneal to a so-called “panhandle.” This RNA structure, with its short double-stranded region and 5ʹ-triphosphate moiety, is an activator of the cytoplasmic RNA helicase RIG-I, an important virus sensor of the antiviral type I interferon (IFN) system (26). Ligand-bound RIG-I signals via the adaptor mitochondrial antiviral-signaling protein (MAVS) and the kinases TBK1/IκB kinase ε (IKKε) to eventually activate the ubiquitously expressed transcription factor interferon regulatory factor 3 (IRF3) (27). The latter thereby becomes phosphorylated, dimerizes, and accumulates in the nucleus, where, together with NF-κB and ATF-2/c-Jun, it transactivates the IFN-β promoter to kick off a first wave of IFN secretion (28). Autocrine and paracrine action of IFN-β then triggers the upregulation of IRF7, which amplifies and diversifies the initial IRF3-driven IFN response by inducing both the *IFNB* gene and multiple *IFNA* genes (29–31). Simultaneously, it induces the transcription of IFN-stimulated genes (ISGs), several of them with demonstrated antiphleboviral activity (3).

Phleboviruses counteract the induction of the IFN response by means of their NSs protein (3, 32). The best-characterized NSs, namely, that of RVFV, allows the full RIG-I signaling cascade to reach the point of IRF3 binding to the IFN-β promoter but then abrogates host gene expression by targeted sequestration and deletion of general transcription factors, as well as by the recruitment of corepressors and induction of an mRNA export block (33–38). In the case of TOSV, in contrast, the NSs protein causes proteasomal degradation of RIG-I (39), and for SFTSV, the NSs sequesters multiple factors of the signaling cascade into cytoplasmic aggregates (40–43). For many phleboviruses, including the sandfly-borne SFSV, however, the mechanism of NSs action is unclear.

We and others previously found that the NSs of SFSV, expressed by a recombinant RVFV, was able to block transcription of the *IFNB* gene (44, 45). Here, we investigated the molecular mechanism and identified IRF3 as a functional target.

## RESULTS

### SFSV NSs inhibits IFN induction.

**FIG 1:**
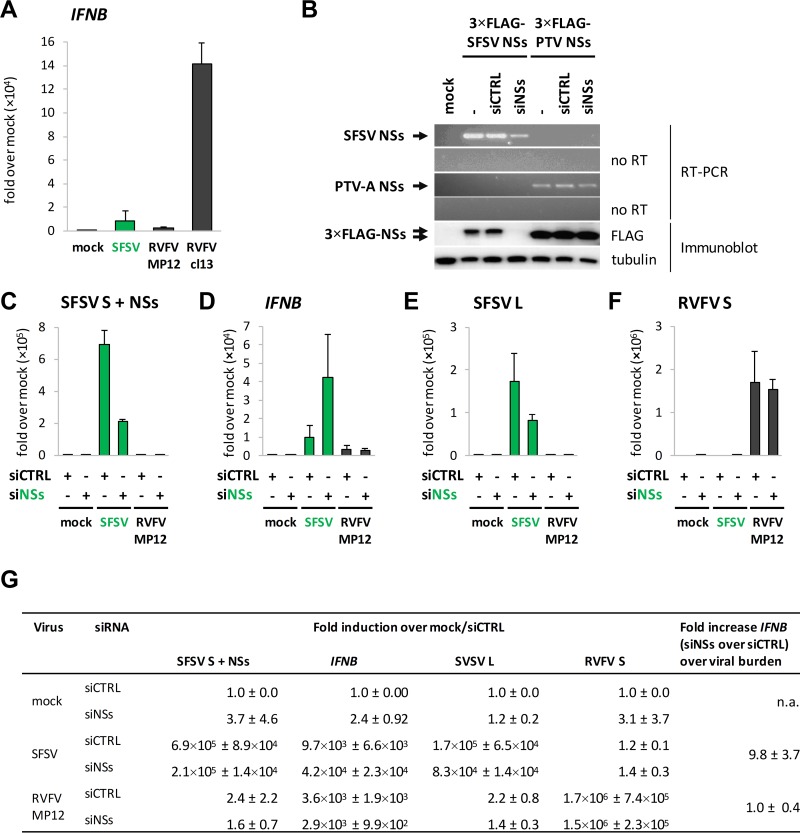
SFSV NSs and *IFNB* induction. (A) A549 cells were infected with SFSV, RVFV MP12, or clone 13 (Cl13) at an MOI of 1, harvested 12 hpi, and analyzed by RT-qPCR analysis for *IFNB* (*n* = 4; mean ± SD). (B) A549 cells were cotransfected with expression constructs for 3×FLAG-tagged SFSV or PTV-A NSs and nontargeting control siRNA or SFSV NSs-specific siRNA. Samples were subjected to RT-PCR analysis (upper panels) and immunoblotting using anti-FLAG and anti-tubulin antibodies (lower panel) 24 h after transfection. To exclude amplification of NSs sequences from plasmid DNA, a duplicate set of reactions was performed without the reverse transcription step (no RT). (C to F) A549 cells were pretransfected with control or SFSV NSs-targeting siRNA and infected with SFSV or RVFV MP12 at an MOI of 1. RNA was isolated 12 hpi for RT-qPCR analysis for NSs-containing RNA (C), *IFNB* (D), the L segment of SFSV (E), and the S segment of RVFV MP12 (*n* = 3; means ± SD) (F). (G) Summary of the relative fold induction data depicted in panels C to F, normalized to the mock sample pretreated with control siRNA as well as the fold induction of *IFNB* in siNSs-treated cells over siCTRL-treated cells that occurred in a manner independent of the viral burden (means ± SD). n.a., not applicable.

SFSV NSs expressed by recombinant RVFV was previously shown to inhibit the upregulation of the *IFNB* gene (44, 45). Accordingly, infection with parental SFSV strain Sabin resulted in only limited upregulation of IFN-β mRNA, as measured by reverse transcriptase quantitative PCR (RT-qPCR) (Fig. 1A). As controls, we used RVFV strain MP12 (expressing a functional RVFV NSs) and clone 13 (expressing an internally deleted RVFV NSs) in parallel (33), which suppressed and activated IFN induction, respectively, in the expected manner.

Unlike RVFV, neither a natural nor a recombinant NSs-deficient strain is available for SFSV. In order to abort NSs function, we designed a pool of four small interfering RNAs (siRNAs) that specifically target the NSs gene sequence. The efficiency of the siRNAs was tested by cotransfection of an expression plasmid for 3×FLAG-tagged SFSV NSs and either the NSs-targeting siRNA pool or a control siRNA. The specific siRNAs caused a significant reduction of SFSV NSs RNA levels in RT-PCR and a complete loss of the FLAG signal in immunoblot analysis, while the control siRNA had no effect (Fig. 1B). In contrast, RNA and protein levels of the 3×FLAG-tagged NSs of PTV-A were not affected, confirming the specificity of the siRNA pool for SFSV NSs.

We then combined transfection of the NSs-specific siRNA pool with infection by either SFSV or RVFV MP-12, followed by RT-qPCR analysis. Of note, in infected cells the siRNA pool as well as the PCR primers can target not only the NSs transcript but also the entire S genome segment. Therefore, we could not determine whether only the NSs mRNA was affected by the siRNAs or whether the viral genome was also affected. However, due to encapsidation of the genome, we expect a certain level of protection, which in turn would result in an underestimation of siRNA effects on NSs transcripts. In any case, a substantial depletion of NSs sequence-containing RNA species (fold reduction, 3.3 ± 0.3) was observed (Fig. 1C). Moreover, in the presence of the NSs-specific siRNAs, SFSV infection upregulated the amounts of IFN-β transcripts (fold increase, 5.1 ± 2.6) (Fig. 1D), despite the fact that virus replication (measured via analysis of L segment levels) was diminished (fold reduction, 2.0 ± 0.42) (Fig. 1E). For RVFV MP12, in contrast, the SFSV NSs-specific siRNAs affected neither the IFN-β mRNA levels (Fig. 1C) nor the accumulation of its S segment (Fig. 1F). The same applied to clone 13, TOSV, and the closely related sandfly fever Turkey virus (data not shown), demonstrating both the specificity of the siRNA pool and its effect on the induction of IFN-β by SFSV. Furthermore, no intrinsic IFN-stimulatory activity of the siRNA pool was observed in the mock samples (Fig. 1C). Taking into consideration the opposing effects of the siRNA on *IFNB* induction and on SFSV replication, a normalized fold induction of 9.8 ± 3.7 was calculated for *IFNB*, compared to 1.0 ± 0.4 for RVFV (Fig. 1G, right column).

Of note, the impairment of SFSV replication by the NSs-specific siRNA was far less pronounced in IFN-incompetent Vero B4 cells (data now shown), indicating that it was largely mediated by the antiviral IFN system rather than by interference with the integrity of the genomic S segment. In summary, siRNA knockdown of SFSV NSs resulted in simultaneous upregulation of IFN induction and downregulation of SFSV replication in IFN-competent cells, reminiscent of the behavior of NSs-deficient phleboviruses. Together with the data from recombinant NSs-expressing RVFV (44, 45), this validates the identification of SFSV NSs as an IFN induction antagonist.

### SFSV NSs acts in a nondegradative manner.

**FIG 2:**
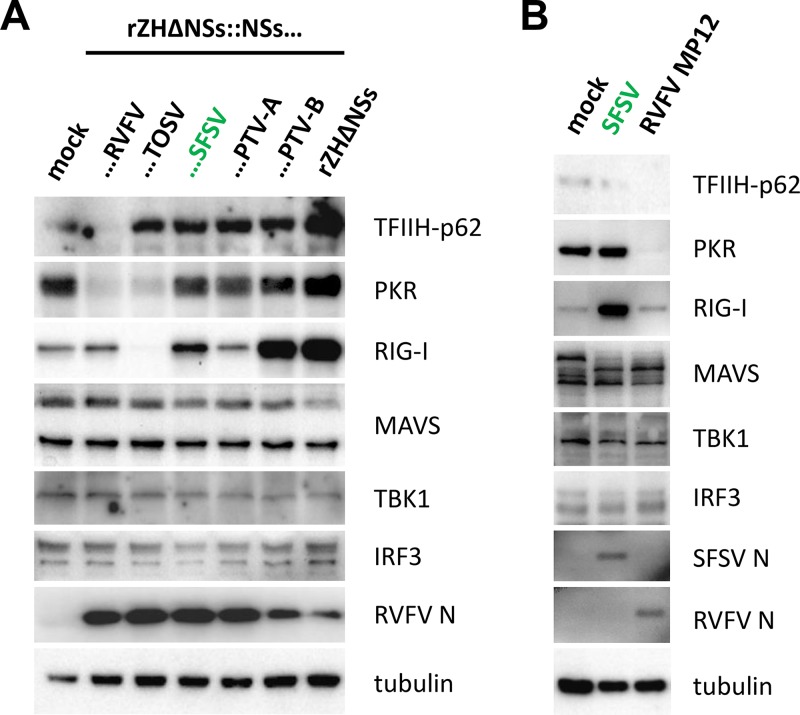
Effect of selected NSs proteins on phlebovirus host targets and central RIG-I signaling components. (A) A549 cells were infected at an MOI of 1 with recombinant RVFV expressing the NSs of RVFV, TOSV, SFSV, PTV-A, or PTV-B or entirely lacking an NSs-coding sequence. Cells were harvested 8 hpi for immunoblot analysis. (B) A549 cells were infected with SFSV or RVFV MP12 at an MOI of 1 and harvested 12 hpi for SDS-PAGE and Western blot analysis.

Many pathogenic phleboviruses are known to counteract the IFN response by diminishing the levels of key host factors (3). The NSs of RVFV induces proteasomal degradation of cellular proteins such as TFIIH-p62 (to block IFN induction) and protein kinase R (PKR) (to prevent the antiviral action of IFN) (35, 44, 46–48). The NSs of TOSV was also shown to cause PKR degradation and to block IFN induction by decreasing RIG-I levels (39, 49). We investigated whether the NSs protein of SFSV might execute a similar form of degradative activity on host proteins. As controls, we employed TOSV NSs and RVFV NSs, and we also included the so far little-investigated PTV NSs, which is known to inhibit host cell transcription (45). For PTV, there are two distinct strains, namely, Adames (PTV-A) and Balliett (PTV-B), which strongly and weakly suppress IFN induction, respectively (45, 50). To directly compare the degradative capacities of the NSs proteins of RVFV, TOSV, SFSV, PTV-A, and PTV-B, we infected A549 cells with recombinant RVFV encoding the respective NSs genes and monitored the intracellular levels of the known phleboviral targets TFIIH-p62, PKR, and RIG-I, as well as of the central RIG-I signaling factors MAVS, TBK1, and IRF3. As shown in Fig 2A, levels of TFIIH-p62 were reduced only by RVFV NSs. Moreover, and in agreement with previous studies (44, 45, 49), PKR levels were decreased upon expression of the NSs of RVFV and TOSV but not by those of SFSV and PTV. RIG-I levels were left unchanged by the NSs of RVFV or PTV-A, strongly decreased by the NSs of TOSV, and upregulated after infection with the recombinant RVFV expressing NSs of SFSV (weakly) or PTV-B (strongly). In fact, in the presence of PTV-B NSs the upregulation of RIG-I was indistinguishable from the level seen with the NSs-deficient control virus rZHΔNSs. The levels of MAVS, TBK1, and IRF3 were not affected by any of the NSs proteins. These results were confirmed in cells infected with the parental SFSV strain Sabin (Fig. 2B). Thus, the NSs proteins of SFSV and PTV do not degrade the host targets of other phleboviruses.

### SFSV NSs inhibits the IRF branch of IFN induction.

**FIG 3:**
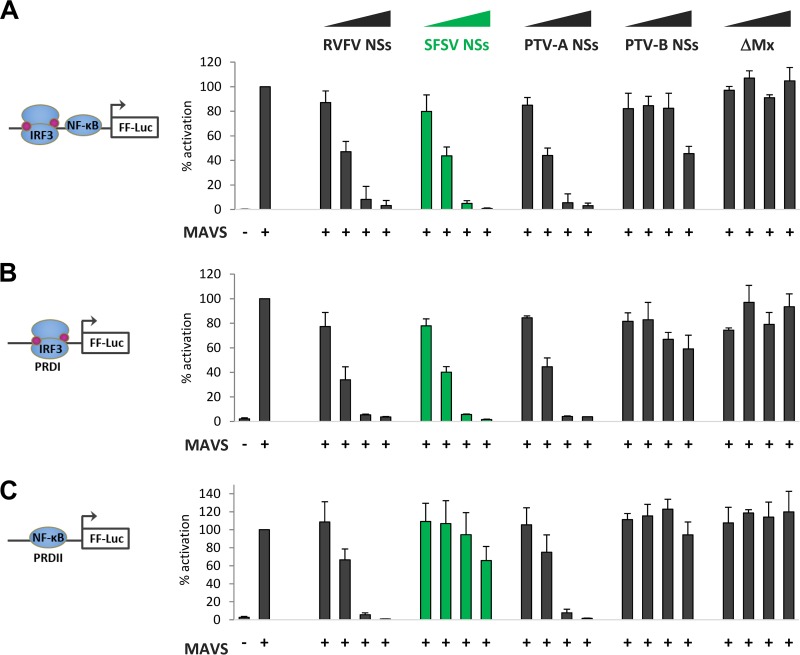
Influence of phlebovirus NSs proteins on *IFNB* promoter elements. HEK293 cells were transfected with expression plasmids for MAVS; NSs of RVFV, SFSV, PTV-A, or PTV-B; or inactive control ΔMx (0.1 ng, 1 ng, or 10 ng), as well as stimulation-dependent firefly luciferase (FF-Luc) and constitutively active *Renilla* luciferase reporters. Firefly luciferase was under the control of (A) the entire IFN-β promoter (*n* = 3; means ± SD), (B) IRF-driven PRDI (*n* = 3; means ±SD), or (C) NF-κB-driven PRDII (*n* = 3; means ± SD). Cell lysates were harvested 24 h after transfection for dual-luciferase assays. Firefly reporter activities were normalized to the *Renilla* reporter activities, and the positive controls were set to 100% prior to calculating means and SD across biological replicates.

For our further investigations, we focused on the NSs of SFSV but also included those of RVFV (as a well-characterized control) and PTV. To interrogate their activity on IFN induction, we performed luciferase reporter assays. Human HEK293 cells were transfected with increasing amounts of expression plasmids encoding the respective NSs proteins, along with a reporter construct harboring the firefly luciferase (FF-Luc) gene under the control of the IFN-β promoter and a constitutively expressing *Renilla* luciferase (R-Luc) plasmid for normalization. Activation of the IFN-β promoter was stimulated by cotransfection of a MAVS cDNA plasmid. As expected, overexpression of MAVS strongly activated the IFN-β promoter, which was undisturbed by increasing doses of the N terminus of the human MxA protein (ΔMx [35]) which was used as a negative control (Fig. 3A). Expression of the NSs proteins of RVFV, SFSV, and PTV-A, in contrast, suppressed the promoter in a dose-dependent manner. PTV-B NSs showed only a partial effect in response to large plasmid amounts, in line with previous observations (50).

The IFN-β promoter contains several positive regulatory domains (PRDs), among which PRDI binds transcription factors of the interferon regulatory factor (IRF) family and PRDII binds NF-κB (51, 52). Reporter assays showed that the inhibitory effect of SFSV NSs on the PRDI promoter element was comparable to that seen with the full IFN-β promoter but that PRDII activity was inhibited only weakly (Fig. 3B and C). This is in contrast to the NSs of PTV-A, which, like the RVFV NSs, inhibited the two PRD reporters indiscriminately. As similar results were obtained when TBK1 was used for stimulation instead of MAVS (data not shown), we concluded that SFSV NSs specifically targets the IRF branch of IFN induction at the level of TBK1 or further downstream, whereas PTV-A NSs blocks IFN induction in a broad manner, as shown previously (45).

### SFSV NSs interacts with IRF3 in a highly specific manner.

**FIG 4:**
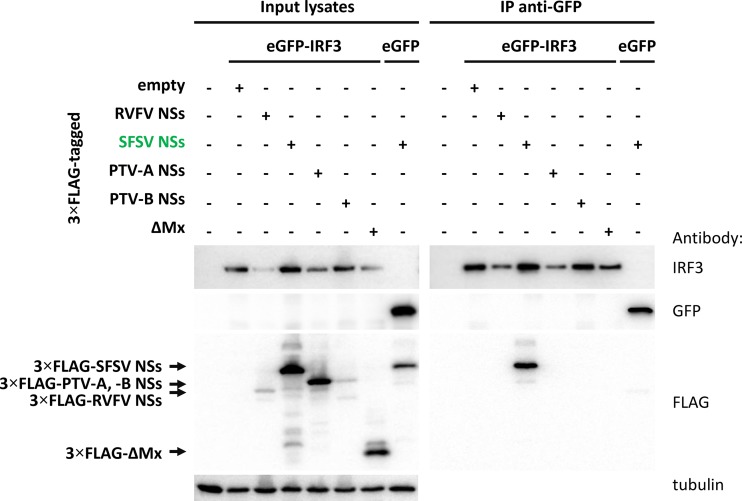
Coimmunoprecipitation of NSs proteins with eGFP-IRF3. Selected 3×FLAG-tagged NSs proteins were coexpressed with eGFP-IRF3 in HEK293 cells. eGFP and ΔMx served as negative controls for eGFP-IRF3 and the NSs proteins, respectively. Cell lysates were subjected to immunoprecipitation via a GFP-binding nanobody immobilized on the bottom of a 96-well plate. Input samples and bound proteins were analyzed via immunoblotting (*n* = 3).

Previously, we took part in a large proteomics screen to identify host cell interactors of viral IFN antagonists that included SFSV NSs (53). The SFSV NSs cDNA, equipped with the sequence for a C-terminal tandem affinity purification (TAP) tag, was inserted into recombinant RVFV to replace the RVFV NSs gene (rRVFVΔNSs::NSs_SFSV_-CTAP). 293T cells were infected with this recombinant virus, tandem affinity purification was performed, and protein complexes were analyzed by liquid chromatography-mass spectrometry (LC-MS). Strikingly, IRF3 was among the host cell interactors of SFSV NSs, which is compatible with the results of our reporter assays. In order to test the data obtained by mass spectrometry, we performed pulldown analyses. An enhanced green fluorescent protein (eGFP)-IRF3 fusion protein was coexpressed with the recombinant 3×FLAG-tagged NSs of SFSV, RVFV, PTV-A, or PTV-B or with the negative-control ΔMx. Cell lysates were then subjected to immunoprecipitation using a plate coated with a nanobody directed against GFP. The NSs proteins of RVFV and PTV-A negatively affected the coexpression of eGFP-IRF3 (Fig. 4 and data not shown), but eGFP-IRF3 was enriched in all GFP precipitates nonetheless. SFSV NSs clearly coprecipitated with eGFP-IRF3 but not with eGFP alone. In contrast, neither of the other phleboviral NSs proteins interacted with eGFP-IRF3. Similar results were also observed in an inverse setting; i.e., SFSV NSs was able to pull down eGFP-IRF3 (or hemagglutinin-IRF3 [HA-IRF3]), while PTV-A and ΔMx were not (data not shown). This confirms our earlier mass spectrometry data (53) and demonstrates that SFSV NSs is unique among the tested phleboviral proteins in its interaction with IRF3.

**FIG 5:**
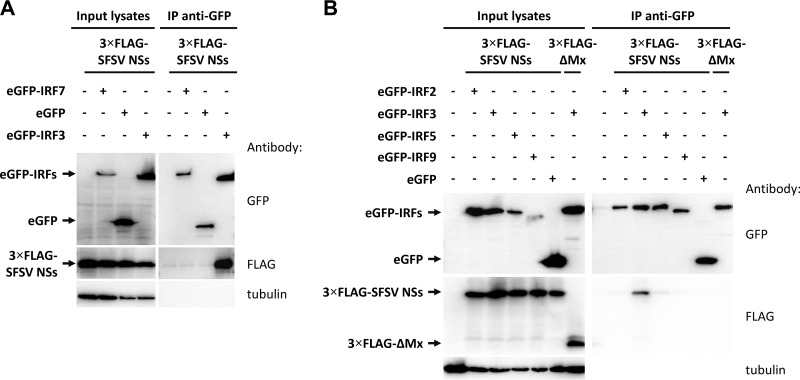
Coimmunoprecipitation of SFSV NSs with IRF proteins. 3×FLAG-tagged SFSV NSs plasmids were transfected into HEK293 cells together with eGFP-fused IRF7 (A) or IRF2, IRF5, or IRF9 (B). eGFP-IRF3 and eGFP were included as positive and negative controls, respectively. Cells were lysed, immunoprecipitation (IP) was performed via the use of GFP, and input lysates and immunoprecipitates were subjected to immunoblotting.

We extended our assays to include other members of the IRF family. IRF7 is the family member most closely related to IRF3 in both sequence and function (31). However, SFSV NSs did not coprecipitate with eGFP-IRF7 (Fig. 5A). Likewise, eGFP-IRF2, eGFP-IRF5, and eGFP-IRF9 did not interact with SFSV NSs (Fig. 5B). Hence, we conclude that SFSV NSs selectively targets the immediate early-acting IFN transcription factor IRF3.

### SFSV NSs does not inhibit IRF3 activation.

**FIG 6:**
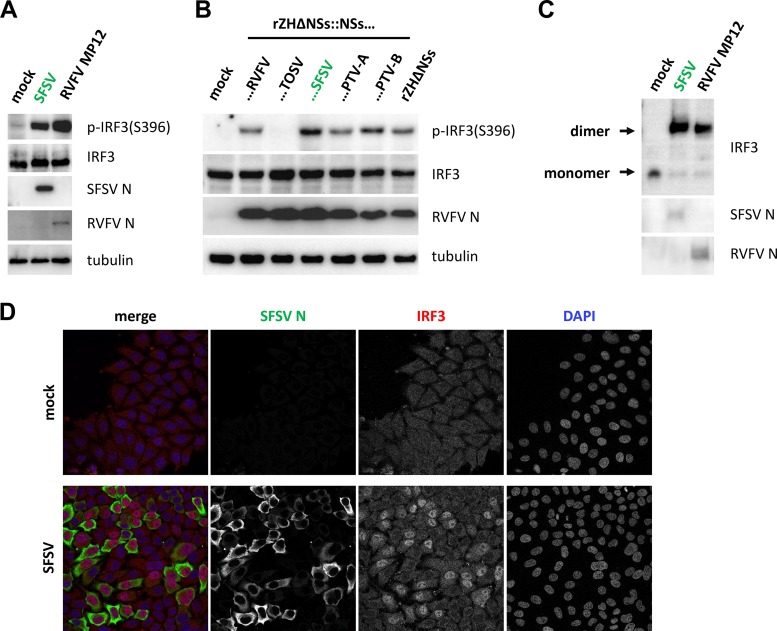
Markers of IRF3 activation under conditions of SFSV infection. (A to C) IRF3 phosphorylation and dimerization. A549 cells were infected with the indicated viruses at an MOI of 1, harvested 12 hpi (A) and 8 hpi (B), and analyzed by immunoblotting for IRF3 phosphorylation and viral nucleocapsid proteins. (C) Samples from the experiment described for panel A were additionally subjected to native PAGE, followed by immunoblotting. (D) Nuclear importation of IRF3. A549 cells seeded onto glass coverslips were infected at an MOI of 1, fixed 12 hpi with paraformaldehyde, and subsequently stained for IRF3 and the SFSV nucleocapsid protein N.

In uninfected cells, IRF3 localizes predominantly to the cytoplasm. Upon activation, IRF3 becomes phosphorylated by TBK1/IKKε, dimerizes, and accumulates in the nucleus, where it associates with the transcriptional cofactors CBP and p300 (52, 54–57). Transiently expressed SFSV NSs, on the other hand, localized diffusely to both the cytoplasm and the nucleoplasm (data not shown), suggesting that it could interfere with IRF3 activation or function at any level. We thus simultaneously investigated the three classic hallmarks of IRF3 activation in SFSV-infected cells. First, immunoblot analysis showed that IRF3 phosphorylation was affected neither in SFSV-infected cells (Fig. 6A) nor in cells infected with a recombinant RVFV expressing SFSV NSs (Fig. 6B). The latter experiment also demonstrated that PTV NSs was acting downstream of IRF3 phosphorylation. Also, IRF3 dimerization (Fig. 6C) and virus-triggered accumulation in the nucleus (Fig. 6D) were not impaired by SFSV infection. Thus, SFSV—like RVFV, which was used as a control (33)—was not preventing phosphorylation, dimerization, or nuclear localization of IRF3.

**FIG 7:**
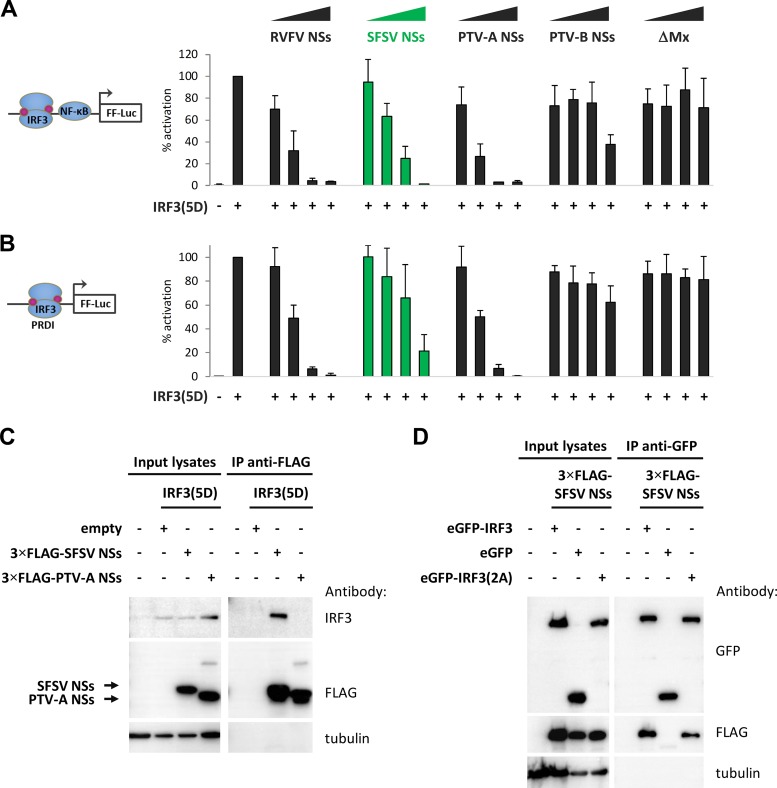
Phosphomimetic and dimerization-deficient IRF3 mutants. (A and B) Promoter reporter assays were performed under conditions of stimulation with a phosphomimetic, constitutively active IRF3(5D). (A) HEK293 cells were transfected with expression plasmids for NSs of RVFV, SFSV, PTV-A, or PTV-B or inactive control ΔMx, as well as firefly and *Renilla* luciferase reporters, under the control of the IFN-β and constitutively active simian virus 40 (SV40) promoters, respectively. IFN-β induction was stimulated by overexpression of IRF3(5D) and total plasmid adjusted to equal levels with empty vector. Firefly activities were normalized to those of *Renilla*, and the stimulation control was set to 100% (*n* = 3; means ± SD). (B) A dual-luciferase assay was performed in parallel with a PRDI-responsive firefly luciferase reporter (*n* = 3; means ± SD). (C and D) Interaction with IRF3 mutants. (C) 3×FLAG-tagged SFSV NSs or PTV-A NSs was coexpressed with IRF3(5D) in HEK293 cells. Cell lysates were then subjected to immunoprecipitation using an antibody against FLAG that was covalently coupled to magnetic beads beforehand. (D) GFP-IRF3(S385/386A), eGFP-IRF3, or eGFP, as well as 3×FLAG-tagged SFSV NSs, was obtained by transient transfection of HEK293 cells. Immunoprecipitation was performed via the use of GFP.

We tested the impact of SFSV NSs on specific IRF3 mutants. IRF3(5D) is constitutively active and dimerized due to phosphomimetic aspartate residues that replace five serine and threonine phosphorylation sites in the region from amino acid (aa) 395 to aa 407 (54, 55). SFSV NSs was able to inhibit both IFN induction and PRD I activation by IRF3(5D) (Fig. 7A and B), just like the NSs of PTV-A, which was used in parallel. SFSV NSs, however, was additionally able to pull down IRF3(5D) (Fig. 7C). SFSV NSs also interacted with IRF3 mutants that are deficient in dimerization, namely, IRF3(S385A/S386A) (58) (Fig. 7D) as well as IRF3(S385A/S386A-R211A/R213A) and IRF3(S385A/S386A-R285A/H288A/H290A), further derivatives with additional mutations of essential arginine and histidine residues within the dimerization interface (data not shown).

In summary, these experiments demonstrated that SFSV NSs inhibits a molecular step that takes place after the nuclear importation of activated IRF3 but prior to IRF3-driven transcription and that the interaction interface on IRF3 is accessible in both the inactive and the active states.

### SFSV NSs interacts with the DNA-binding domain of IRF3.

**FIG 8:**
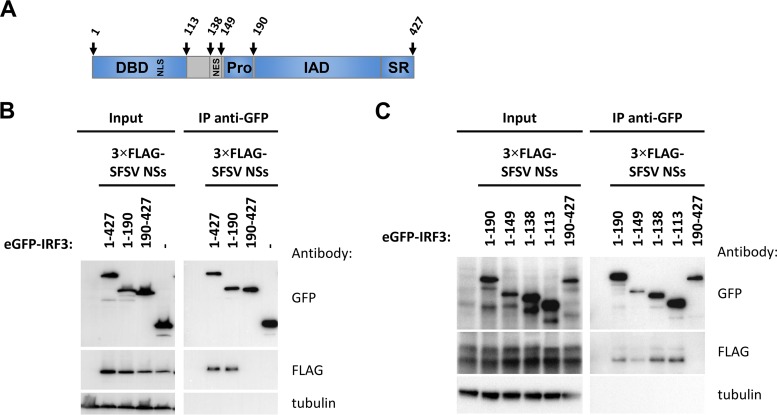
FIG 8 Domain mapping of binding region within IRF3. (A) Schematic representation of the IRF3 domain structure. IRF3 contains a DNA-binding domain (DBD, aa 1 to 113) with an embedded bipartite nuclear localization signal(s) (NLS; K77/R78 and R86/K87), a nuclear export signal (NES; aa 139 to 150), and a proline-rich region (Pro;aa 150 to 190) directly followed by the IRF association domain (IAD; aa 190 to 384) and a serine-rich region (SR; aa 384 to 427) at the C terminus. (B) eGFP-fused full-length IRF3, its N-terminal portion (1–190) or C-terminal portion (190–427), or eGFP alone was expressed together with 3×FLAG-tagged SFSV NSs in HEK293 cells, followed by immunoprecipitation via the use of GFP and immunoblotting. (C) A series of successively truncated eGFP-IRF3 mutants were produced from DNA templates by coupled *in vitro* transcription-translation and added to lysates of HEK293 cells expressing 3×FLAG-tagged SFSV NSs for subsequent immunoprecipitation via the use of GFP.

IRF3 possesses an N-terminal DNA-binding domain (DBD; aa 1 to 113) (59) which also contains the bipartite nuclear localization signal (NLS; K77/R78 and R86/K87) (60, 61), followed by an activation domain comprising the nuclear export signal (NES; aa 139 to 150) (52, 60), a proline-rich domain (Pro; aa 150 to 190), an IRF association domain (IAD; aa 190 to 384) (62), and a serine-rich domain (SR; aa 384 to 427) that is phosphorylated upon activation (63) (Fig. 8A). Crystal structures of the C-terminal portion of IRF3 (aa 173/175 to 427) indicate that IRF3 phosphorylation induces a marked conformational change in the IAD, resulting in the exposure of residues that facilitate dimerization and the interaction with CBP/p300 (58, 64, 65). We employed systematic deletion analysis to map the IRF3 domain that is bound by SFSV NSs. As a first step, we cut GFP-tagged IRF3 into two halves at position 190. As shown in Fig. 8B, only the N-terminal part, ranging from aa 1 to 190, was able to pull down NSs. We then removed the remaining domains from this fragment one by one in the C- to N-terminal direction. In this way, we found that the N-terminal DBD alone (aa 1 to 113) was sufficient for binding SFSV NSs (Fig. 8C). Unfortunately, fine mapping by further C-terminal deletions was inconclusive, as were our attempts to map the corresponding IRF3-interacting region within SFSV NSs (data not shown).

### SFSV NSs prevents IRF3 from binding to the IFN promoter.

**FIG 9:**
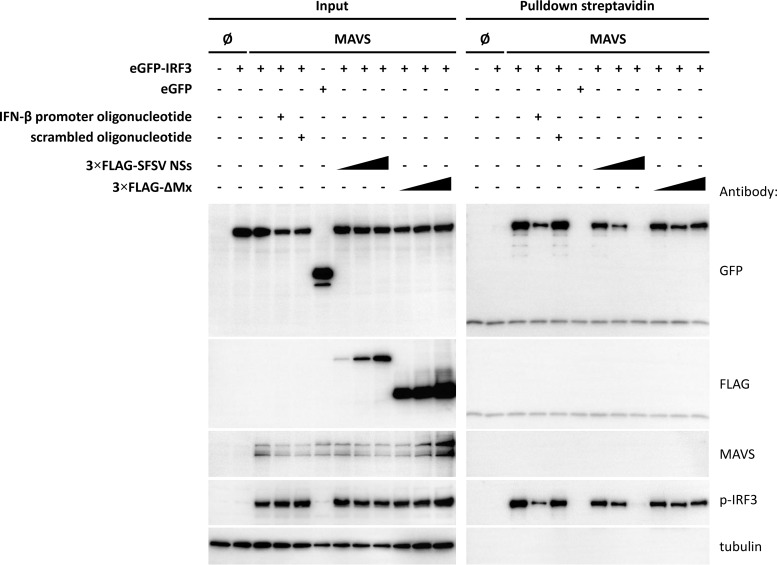
IFN-β promoter binding assay. HEK293 cells were cotransfected with plasmids encoding eGFP-IRF3 or eGFP or MAVS, as well as with increasing amounts of plasmids encoding 3×FLAG-tagged SFSV NSs, or the 3×FLAG-tagged control protein ΔMx, as indicated. Cell lysates were then incubated with an unlabeled, double-stranded DNA oligonucleotide comprising the IFN-β promoter or with a scrambled control oligonucleotide or were left untreated. Next, streptavidin-coated magnetic beads covered with biotinylated IFN-β promoter oligonucleotide were used to pull down activated IRF3. Bound proteins were eluted by boiling in Laemmli buffer and analyzed by immunoblotting.

We hypothesized that SFSV NSs might interfere with the promoter-binding activity of IRF3. To investigate this, we established an assay in which we used biotinylated IFN-β promoter oligonucleotides to pull down MAVS-activated IRF3 via the use of streptavidin-coated magnetic beads. eGFP-IRF3 and MAVS were coexpressed in HEK293 cells either on their own or together with increasing doses of 3×FLAG-tagged SFSV NSs or the negative-control ΔMx. As observable in the input samples, overexpressed MAVS induced the phosphorylation and dimerization of eGFP-IRF3, as expected (Fig. 9, left panels, and data not shown). The presence of SFSV NSs did not affect IRF3 activation, confirming our observations of SFSV-infected cells. Analyzing the precipitated proteins (Fig. 9, right panels), we detected activated eGFP-IRF3 but not eGFP, indicating specific binding to the IFN-β promoter oligonucleotide. Furthermore, no protein precipitation was observed when empty beads without the biotinylated oligonucleotide were used (data not shown). The sequence specificity of eGFP-IRF3 binding was confirmed by the addition of an excess of nonbiotinylated IFN-β promoter oligonucleotide, which strongly diminished eGFP-IRF3 binding, whereas a scrambled control oligonucleotide had no such effect. Importantly, coexpression of SFSV NSs reduced the amount of promoter-bound eGFP-IRF3 in a dose-dependent manner, but the control protein ΔMx had no influence. Of note, SFSV NSs did not coprecipitate with the promoter oligonucleotide, indicating the absence of intrinsic or indirect DNA-binding activity. Thus, we conclude that SFSV NSs stoichiometrically impairs the binding of IRF3 to the IFN-β promoter by covering essential amino acid residues within the DBD.

## DISCUSSION

SFSV, first isolated in 1943 (11), is one of the geographically most widespread members of the genus phlebovirus, with high seroprevalence rates in regions of endemicity (12–20). Despite the long-standing association with an acute incapacitating disease, little is known about the interaction of SFSV with the host cell. Also, there is no established animal model (11, 24), prompting earlier researchers to fall back on experiments with human volunteers (9).

We found that the induction of IFN-β in SFSV-infected cells is inhibited by NSs, although SFSV does not destroy any of the key antiviral host factors that other dipteran-borne phleboviruses attack. Rather, SFSV NSs binds to the DBD of IRF3, thus prohibiting IFN-β promoter activation. Curiously, none of the other IRF family members, including the master regulator IRF7 (66), are targeted, indicating high specificity. Although a significant role in the generation of a full IFN response has been attributed to the IFN-inducible IRF7 (66), the constitutively expressed IRF3 is indispensable for the induction of a first wave of IFN-β expression from virus-infected cells and the subsequent upregulation of IRF7 expression (30, 31). Hence, *Irf3* knockout mice exhibit substantially increased susceptibility to viral infection (31, 67). IRF3 activation is the target of a number of virulence factors (28), e.g., human papillomavirus 16 (HPV16) E6 (68), the V protein of paramyxoviruses (69), herpes simplex virus 1 (HSV-1) VP16 (70), and rotavirus NSP1 (71). However, in contrast to SFSV NSs, these virulence factors affect phosphorylation, dimerization, nuclear accumulation, or the expression level of IRF3. SFSV NSs interacted with nonactivated and constitutively active (and dimerized) as well as dimerization-incompetent IRF3, suggesting an ability to target IRF3 both before and after it becomes activated. Moreover, this interaction pattern pointed to a region of IRF3 that is accessible independently of its activation and dimerization state. Domain mapping consistently revealed that the N-terminal DBD alone was sufficient for binding of SFSV NSs. Taking the data together, including the interference with SFSV NSs at a late stage in the signaling pathway on the one hand and the domain mapping on the other hand, a mechanism involving the sequestration of the DBD by SFSV NSs from the IFN-β promoter was strongly implied, and its presence was confirmed by a promoter binding assay.

The N-terminal DNA-binding domain of interferon regulatory factors is about 120 amino acid residues long and displays a conserved architecture consisting of three α helices, four β sheets, and three loops (L1 to L3) in the order α1-β1-β2-L1-α2-L2-α3-β3-L3-β4 (51). As SFSV NSs (i) interferes with the promoter binding activity of IRF3 but (ii) does not interact with other IRF family members, one could speculate that it targets amino acid residues that are involved in DNA binding but that are not conserved within the IRF family. IRF3 residues L42, R78, and R86 are both nonconserved and involved in specific DNA promoter binding (51). However, R78 and R86 are also part of the bipartite IRF3 NLS. Since SFSV NSs does not interfere with the nuclear importation of IRF3, these residues are less likely to mediate the interaction with SFSV NSs. That leaves DNA binding residue L42 (situated in loop L1) as well as less-conserved strands β3 and β4 and loops L2 and L3 as the most probable candidate binding sites for SFSV NSs.

Among the other viral proteins known to target IRF3, only US1 (also ICP22) of herpes simplex virus 2 and NP1 of human bocavirus have been described to employ a similar mechanism (72, 73). Like SFSV, both these viruses target the IRF3 DBD and disrupt promoter binding, but whether this is restricted to IRF3 or also true for any other member of the IRF family was not addressed. Kaposi’s sarcoma-associated herpesvirus proteins K-bZIP and LANA-1 also prevent the binding of activated IRF3 to its cognate promoter sites but do so by occupying the promoter sites themselves (74, 75), which we did not observe for SFSV NSs. In addition to these DNA viruses, bovine viral diarrhea virus interferes with promoter binding and then induces the degradation of nuclear IRF3 via its NPro protein (76, 77). A direct interaction between NPro and IRF3 could not be demonstrated, however. Hence, to our knowledge SFSV NSs seems to be the only virulence factor from an RNA virus which acts by directly masking the DBD of IRF3 to prevent promoter binding and IFN induction.

Given the remarkable diversity of the phleboviral NSs proteins with respect to sequences, subcellular localizations, and molecular mechanisms, it is tempting to speculate on a correlation between the specific anti-IFN strategy of a given NSs protein and the degree of virulence of the respective phlebovirus. The NSs of highly virulent RVFV uses multiple strategies, mostly based on proteasomal degradation, to globally and rapidly blunt host gene expression at the transcriptional and posttranscriptional levels (3, 32). The NSs of the highly virulent SFTSV abrogates IFN induction by sequestering several key signaling components, including RIG-I and TBK1, into cytoplasmic aggregates (40–43). The intermediately pathogenic TOSV acts by degrading RIG-I itself (39), but its NSs seems to be degraded along with its host target, cutting down its inhibitory efficiency (78). The NSs of the apathogenic Uukuniemi virus (UUKV), in contrast, does not significantly inhibit IFN induction (79, 80). How does the intermediately pathogenic SFSV fit into this picture? On the one hand, by masking the DBD to sterically hinder IRF3 from binding the IFN-β promoter, SFSV NSs blocks IRF3 independently of its conformation or activation state. On the other hand, however, this stoichiometric mechanism requires NSs to accumulate to levels that are sufficient for sequestering the cellular pool of IRF3, which, during the early phase of infection, outnumbers NSs. Moreover, SFSV NSs inhibition does not include IRF7, the master regulator of innate immunity (66). Thus, SFSV NSs fail to impair IFN induction in cells where upregulation of IRF7 took place before infection or in cells with physiologically high basic levels of IRF7, such as plasmacytoid dendritic cells (81). In other words, the stoichiometric and IRF3-specific nature of the anti-IFN induction strategy makes SFSV NSs a modulator rather than a full antagonist of IFN induction. This places SFSV between TOSV and UUKV with regard to both anti-IFN strategy (RIG-I degradation versus weak IFN antagonism) and virulence (fever and meningitis/encephalitis versus no disease).

Curiously, PTV-A does not seem to quite fit the picture; while its NSs protein seems to act as a global host transcription inhibitor, infection of humans has so far been associated only with febrile symptoms. In rodent models, such as mouse and hamster, however, PTV-A and chimeric RVFVs that express PTV-A NSs are also highly virulent (50, 82, 83), suggesting that PTV may be an outlier with respect to humans but not other mammals. Thus, the demonstration that the intermediately virulent SFSV specifically targets IRF3 in a highly specific and stoichiometric (i.e. nondestructive) manner supports our hypothesis that the molecular strategy employed by the NSs protein can correlate with the degree of virulence of the parental phlebovirus, although other factors, e.g., cell tropism, RNA polymerase activity, species-specific host protein interactions, and escape from adaptive immunity, are of course equally important.

## MATERIALS AND METHODS

### Cells, viruses, and plasmids.

A549, BHK-21, HEK293, HEK293T, Vero B4, and Vero E6 cells were cultured in Dulbecco’s minimal essential medium (DMEM) and CCM34 medium (DMEM with addition of 17.8 mg/liter l-alanine, 0.7 g/liter glycine, 75 mg/liter l-glutamic acid, 25 mg/liter L-proline, 0.1 mg/liter biotin, 25 mg/liter hypoxanthine, and 3.7 g/liter sodium bicarbonate) supplemented with 10% fetal calf serum (FCS), 2 mM glutamine, 100 U/ml penicillin, and 100 µg/ml streptomycin.

The Sabin strain of SFSV was obtained from the World Reference Center for Emerging Viruses and Arboviruses (WRCEVA) and propagated in Vero B4 cells. Attenuated RVFV strains MP12 and clone 13 were propagated in BHK-21 cells. Recombinant RVFV strains rZH548, rZH548ΔNSs, rZH548ΔNSs::NSsSFSV, and rZH548ΔNSs::NSsTOSV have been described previously (44, 84, 85). rZH548ΔNSs::NSsPTV-A, rZH548ΔNSs::PTV-B, and rZH548ΔNSs::NSsSFSV-CTAP were generated using a polymerase I (Pol I)/Pol II-based rescue system as described for the other recombinant RVFV strains (35, 53, 85). In brief, NSs coding sequences for PTV-A and PTV-B NSs (GenBank accession no. EF201835 and EF201834, respectively) were obtained by gene synthesis (Mr. Gene) and inserted into modified S-segment rescue plasmid pHH21_RVFV_vN_TCS. The reading frame of SFSV NSs was amplified from cDNA of infected cells and inserted into rescue plasmid pHH21_RVFV_vN_MCS_CTAP, which contains a C-terminal tag for tandem affinity purification (TAP). Primer sequences are available on request. The resulting plasmids were transfected together with L- and M-segment rescue plasmids pHH21_RVFV_vL and pHH21_RVFV_vM, respectively, as well as helper plasmids pI.18_RVFV_L and pI.18_RVFV_N into cocultures of HEK293T and BHK-21 cells. Recombinant RVFV strains were harvested 5 days after transfection, propagated in Vero E6 cells, and characterized by RT-PCR and sequencing of the N- and NSs-coding regions. Titers of all virus strains were determined on Vero E6 cells via plaque assay. Both the cell lines and the virus stocks were routinely tested for mycoplasma contamination.

To generate constructs encoding 3×FLAG-tagged NSs of SFSV (GenBank accession no. EF201822.1), PTV-A, or PTV-B, the viral open reading frames were amplified from cDNA (SFSV) or synthesized DNA (PTV-A and PTV-B) and inserted into pI.18 by ligation-dependent cloning via the use of 5ʹ BamHI and 3ʹ XhoI restriction sites. Primer sequences are available on request. pI.18-NSsRVFV-3×FLAG and pI.18-3×FLAG-ΔMx were described before (35). Firefly luciferase reporter constructs p-125Luc, p-55C1BLuc, and p-55A2Luc (52) were kindly donated by Takashi Fujita, and pRL-SV40 was purchased from Promega. Expression plasmids for human TBK1 (86) and IRF3(5D) (55) were kindly provided by John Hiscott, for human MAVS by Shizuo Akira (87), and for full-length pEGFP-C1-IRF3 (88) and all other pEGFP-C1-IRFs by Luis Martinez-Sobrido and Adolfo Garcia-Sastre. pEGFP-C1 was from Clontech. pcDNA3.1(+)-eGFP-IRF7, pEGFP-C1-IRF3(1-190), pEGFP-C1-IRF3(190-427), pEGFP-C1-IRF3(385A/S386A), pEGFP-C1-IRF3(S385A/S386A-R211A/R213A), and pEGFP-C1-IRF3(S385A/S386A-R285A/H288A/H290A) were generated via gene synthesis and subcloning (BioCat and Eurofins Genomics).

### siRNA-mediated knockdown and infection.

Reverse transfection of A549 cells (1 × 10^5^ per 24-well) with either control siRNA (1027280; Qiagen) or a pool of four custom-designed siRNA oligonucleotides targeting SFSV NSs (siNSs1 [5ʹ-TTG GGT CTT AGT GAT GAG CAT-3ʹ], siNSs2 [5ʹ-AAG GGA TCA GCT AAT GTC TTA-3ʹ], siNSs3 [5ʹ-TAC AAT AAA TTT CAC ACT CAT-3ʹ], and siNSs4 [5ʹ-AAG GCT CTT AGC TGG CCA CTA-3ʹ]; Qiagen) via the use of Lipofectamine RNAiMax (Life Technologies) was performed according to the manufacturer's recommendations. Cells were washed with sterile phosphate-buffered saline (PBS) at 24 h posttransfection and inoculated with virus diluted to a multiplicity of infection (MOI) of 1 in CCM34 supplemented with 2% FCS and antibiotics. After 1 h of incubation at 37°C, the inoculate was replaced by CCM34 supplemented with 10% FCS and antibiotics. For concomitant transfection of siRNA and plasmid DNA, Lipofectamine 2000 was used instead.

### Reverse transcriptase PCR (RT-PCR).

RNA was isolated using an RNeasy minikit (Qiagen) as recommended by the manufacturer. RNA from infected cells was subjected to DNase I digestion and cDNA synthesis using a PrimeScript RT reagent kit with genomic DNA (gDNA) Eraser (TaKaRa). Transcript levels of host genes were detected with SYBR Premix Ex *Taq* (Tli RNaseH Plus) (TaKaRa) and QuantiTect primers (human *IFNB*, QT00203763; *RRN18S*, QT00199367; Qiagen), whereas viral genomic segments were detected with Premix Ex *Taq* (Probe qPCR) (TaKaRa) and previously published primers and probes for the SFSV and RVFV S and L segments (for SFSV S, fwd, 5ʹ-TGC ACT CAT CCA AGC TAT GTG-3ʹ, rev, 5ʹ-GAG GGC TAC AAA CAA GGG ATC-3ʹ, probe, 6-carboxyfluorescein [FAM]-TCC CCC ATT CTC AGA ATG TAA GAC ATT AGC-black hole quencher 1 [BHQ-1] [89]; for SFSV L, fwd, 5ʹ-TCT GAG AAC TGA GCT ACA AGT GTT TAT TA-3ʹ, rev, 5ʹ-TTC CCA TCT CTC TTC TGA AGA GTG-3ʹ, probe, FAM-AGG TCA TAG ACA GTA TCA TGA GAA TTG CTA GGT G-BHQ-1 [4]; for RVFV S, fwd, 5ʹ-TGC CAC GAG TYA GAG CCA-3ʹ, rev, 5ʹ-GTG GGT CCG AGA GTY TGC-3ʹ, probe, FAM-TCC TTC TCC CAG TCA GCC CCA C-BHQ-1 [89]). Fold induction was calculated according to the threshold cycle (ΔΔ*C_T_*) method using 18S rRNA as a housekeeping gene.

RNA from transfected cells was subjected to DNase I digestion (Fermentas), and NSs transcripts were amplified via OneStep RT-PCR (Qiagen) according to the manufacturer's instructions (for SFSV NSs, fwd, 5ʹ-ATA TGG ATC CAT GAA CAG CCA GTA CAT GTT-3ʹ, rev, 5ʹ-GAC ACT CGA GTC AAA AGT CAG AGT CAG ACG-3ʹ; for PTV-A NSs, fwd, 5ʹ-GAG AGG ATC CAT GTC CAA CAT AAA CTA TTA TG-3ʹ, rev, 5ʹ-GAC ACT CGA GTT ATA TGT CTT GAT TTA GCA TTG-3ʹ). Amplification products were run on 1.5% agarose gels and visualized with ethidium bromide.

### Immunoblot analysis.

Protein samples were run on 12% acrylamide gels and transferred to polyvinylidene fluoride (PVDF) membranes (Millipore) via semidry blotting. After blocking in Tris-buffered saline (TBS) with 5% bovine serum albumin (BSA) or milk powder, primary antibody staining was performed for 1 h at room temperature or overnight at 4°C. Membranes were washed in TBS–0.1% Tween 20, stained with secondary antibodies for 45 min, and washed again in TBS–0.1% Tween 20 and once in TBS. Finally, membranes were developed with a SuperSignal West Femto kit (Pierce) and bands visualized using a ChemiDoc imaging system (Bio-Rad).

Primary antibodies were as follows: RIG-I (ag-20b-0009; AdipoGen) (1:1,000), MAVS (ALX-210-929; Alexis) (1:1,000), TBK1 (IMG-139A; Imgenex) (1:1,000), PKR (610764; BD Transduction Laboratories) (1:1,000), IRF3 (sc-9082; Santa Cruz) (1:500), p62 (ab55199; Abcam) (1:2,000), GFP (3h9; Chromotek) (1:2,000), FLAG (F3165; Sigma) (1:2,000), p-IRF3 (catalog no. 4947; Cell Signaling) (1:1,000), tubulin (ab6046; Abcam) (1:2,500), SFSV N (mouse immune ascites fluid, provided by WRCEVA) (1:1,000), and RVFV N (rabbit hyperimmune serum, provided by Alenjandro Brun) (1:1,000). Secondary antibodies comprised anti-mouse (0031430 1892913; Thermo Fisher), anti-rabbit (0031460 1892914; Thermo Fisher), and anti-rat (712-036-150; Jackson Immuno Research) antibodies or were substituted by protein A horseradish peroxidase (HRP) conjugate (18-160; Millipore) (1:10,000).

### Dual-luciferase assay.

HEK293 cells seeded into 96-well plates (1.5 × 10^4^ per well) were transfected the following day with firefly and *Renilla* luciferase reporter constructs (40 ng each), as well as expression constructs for MAVS (10 ng) and NSs proteins or the control protein ΔMx (0.1 ng, 1 ng, and 10 ng) via the use of TransIT-LT1 (Mirus Bio LLC). The total plasmid DNA amounts were adjusted to equal levels with empty vector pI.18. Cells were processed 24 h after transfection, and luciferase activities were measured with a dual-luciferase reporter assay system (Promega) according to the manufacturer’s recommendations. Firefly luciferase activities were normalized to those of *Renilla* luciferase, and the stimulated control samples were set to 100% within each biological replicate. Means and standard deviations (SD) were calculated across the indicated number of biological replicate data sets.

### Proteomics.

As described previously (35, 53), approximately 2 × 10^8^ HEK293T cells were infected with the recombinant RVFV strain expressing TAP-tagged SFSV NSs (rZH548ΔNSs::NSsSFSV-CTAP) at an MOI of 5. The cells were washed with and scraped off in prechilled PBS at 16 h postinfection (hpi). The cell pellet was snap-frozen in liquid nitrogen, lysed in TAP buffer (50 mM Tris-HCl [pH 7.5], 100 mM NaCl, 0.2% NP-40, 5% glycerol) supplemented with protease and phosphatase inhibitors, snap-frozen again, and stored at −80°C until further processing. TAP purification was performed by sequential pulldowns using streptavidin agarose and HA-agarose beads. Bound protein complexes were eventually eluted in Laemmli buffer and subjected to one-dimensional SDS-PAGE prior to trypsin digestion and peptide analysis by liquid chromatography-tandem mass spectrometry (LC-MS/MS), which was described in detail elsewhere (53).

### Coimmunoprecipitation.

HEK293 cells (2.5 **×** 10^6^ per 10-cm-diameter dish) were transfected with expression plasmids (4 µg each) via the calcium phosphate method. Cells were washed twice in PBS the following day and lysed in prechilled lysis buffer (50 mM Tris-HCl [pH 7.0], 150 mM NaCl, 1% IGEPAL-630) freshly supplemented with protease (Roche) (complete, EDTA-free) and phosphatase inhibitors (Phosphatase Inhibitor Cocktail set II; Calbiochem). Finally, cell debris was removed by centrifugation (10,000 × *g*, 10 min, 4°C), and the supernatants were used for further processing.

For immunoprecipitation via the use of GFP, supernatants were applied to prewashed wells of a GFP-multiTrap (Chromotek) and incubated at 4°C for 60 to 90 min under conditions of mild shaking. Wells were washed extensively with lysis buffer and bound proteins eluted for 20 min with preheated Laemmli buffer under conditions of strong agitation. For immunoprecipitation via the use of FLAG, magnetic beads (143-21D; Invitrogen) were covalently coupled with FLAG M2 antibody (F3165; Sigma) overnight and processed according to the manufacturer's recommendations. Lysates were then added to the coupled beads followed by incubation under conditions of rotation at 4°C for 4 h. After extensive washing, bound proteins were eluted by boiling in Laemmli buffer at 94°C for 5 min.

To map the binding region within IRF3, constructs comprising a T7 promoter, the open reading frames (ORF) of the respective truncated IRF3 mutants fused to eGFP, a stop codon, and a poly(A) stretch were assembled via PCR (primer sequences available on request) and purified via gel extraction (Omega Bio-tek) and DNA precipitation. The respective proteins were then produced by coupled *in vitro* transcription-translation using rabbit reticulocyte lysate (L4610; Promega) and added to lysate of HEK293 cells transiently expressing SFSV NSs. Immunoprecipitation via GFP was performed according to the aforementioned protocol.

### IRF3 dimerization assay.

A549 cells infected with SFSV were lysed as described above and then processed as described before (90). In brief, 10% native polyacrylamide gels were prerun at 25 mA for 30 min in native running buffer (25 mM Tris, 192 mM glycine, pH 8.3), with 1% deoxycholate added to the cathode buffer. Samples were supplemented with native loading buffer (250 mM Tris-HCl [pH 6.8], 50% glycerol, 1% deoxycholate, 0.5% bromophenol blue), run at 20 mA for the desired duration, and finally transferred to PVDF membranes via semidry blotting.

### Immunofluorescence assay.

A549 cells were seeded onto glass coverslips (1 × 10^5^ per 24 wells) 1 day prior to infection at an MOI of 1. The cells were washed with PBS at 12 hpi and fixed overnight in PBS–4% paraformaldehyde (PFA) at 4°C. The coverslips were then washed with PBS, and the cells were permeabilized with PBS–0.1% Triton X-100, washed again, and blocked in PBS–1% FCS. Staining with primary antibodies diluted in blocking buffer (IRF3 FL-425, 1:200; SFSV, 1:2,500) was performed for 1 h in a humid chamber. Afterward, the coverslips were washed with PBS and incubated with secondary antibodies (Alexa Fluor 488 donkey anti-mouse [A21202] and Alexa Fluor 647 donkey anti-rabbit [A31573]; Thermo Fisher Scientific) (both 1:500) and 4′,6-diamidino-2-phenylindole (DAPI) (0.1 µg/ml) for 45 min in a humid chamber. Samples were washed again in PBS, rinsed in demineralized water, and mounted on microscopic slides using FluorSave reagent (Calbiochem). Confocal microscopy was performed using a Leica SP5 microscope and the accompanying software.

### Promoter binding assay.

Biotinylated DNA covering the IRF3-responsive positive regulatory domains within the human IFN-β promoter and the downstream sequence as a linker (GenBank accession no. EF064725.1) was ordered as complementary single DNA strands (sense, 5ʹ-GAC ATA GGA AAA CTG AAA GGG AGA AGT GAA AGT GGG AAA TTC CTC TGA ATA GAG AGA GGA CCA TCT CAT ATA AAT AGG CCA TAC CCA TGG AGA AAG GAC ATT-biotin-3ʹ; antisense, 5ʹ-AAT GTC CTT TCT CCA TGG GTA TGG CCT ATT TAT ATG AGA TGG TCC TCT CTC TAT TCA GAG GAA TTT CCC ACT TTC ACT TCT CCC TTT CAG TTT TCC TAT GTC-3ʹ) and subsequently annealed by initial denaturation at 95°C for 5 min, followed by slow cooling (1°C/min) to room temperature (91). The double-stranded biotinylated oligonucleotide (10 pmol per sample) was bound to streptavidin-coated magnetic beads (Dynabeads M-280 streptavidin; Invitrogen) (25 µl per sample) according to the manufacturer's instructions. HEK293 cells seeded into 6 wells (2.5 × 10^5^ per well) were transfected with plasmids coding for eGFP-IRF3 or for eGFP (250 ng), MAVS (500 ng), 3×FLAG-tagged SFSV NSs, or ΔMx (25, 250, or 500 ng) and empty vector (to adjust plasmid amounts) via the use of TransIT-LT1 and lysed in the presence of protease and phosphatase inhibitors as described above. Lysates were then incubated with 250 pmol of the corresponding untagged IFN-β promoter oligonucleotide or 250 pmol of scrambled control oligonucleotide (sense, 5ʹ-TTA CAG GAA AGA GGT ACC CAT ACC GGA TAA ATA TAC TCT ACC AGG AGA GAG ATA AGT CTC CTT AAA GGG TGA AAG TGA AGA GGG AAA GTC AAA AGG ATA CAG-3ʹ; antisense, 5ʹ-CTG TAT CCT TTT GAC TTT CCC TCT TCA CTT TCA CCC TTT AAG GAG ACT TAT CTC TCT CCT GGT AGA GTA TAT TTA TCC GGT ATG GGT ACC TCT TTC CTG TAA-3ʹ) or were left untreated. After addition of oligonucleotide-coupled magnetic beads, samples were incubated under conditions of rotation for 90 min. The beads were washed four times in lysis buffer prior to elution of bound proteins in Laemmli buffer at 94°C for 5 min and analysis via SDS-PAGE and Western blotting.
